# Port-Site Metastasis after Laparoscopic Surgery for Urological Malignancy: Forgotten or Missed

**DOI:** 10.1155/2012/609531

**Published:** 2012-04-10

**Authors:** N. Kadi, M. Isherwood, M. Al-Akraa, S. Williams

**Affiliations:** ^1^The Urology Department, Royal Derby Hospital, Derby, DE22 3NE, UK; ^2^The Urology Department, The Royal Free Hospital, London, NW3 2QG, UK

## Abstract

*Purpose*. Port-site metastasis has been a concern with the common use of laparoscopy in urologic oncology. We conducted this study to provide a review of port-site metastases reported after the laparoscopy in managing urologic malignancies, possible contributing factors and preventative measures. *Materials and Methods.* An electronic search of MEDLINE using the combined MESH key words “port-site metastasis” and “Urology”. *Results*. 51 articles addressing port-site metastasis after laparoscopic surgery for urolo¬gical malignancy were identified. *Conclusion*. Port-site metastasis after laparoscopic surgery for urolo¬gical malignancy is rare. The incidence is comparable to the rate for surgical wound metastases.

## 1. Introduction

In recent years, with the widespread use of laparoscopy to treat an ever-increasing number of urologic malignancies, questions have been raised about the oncologic safety of this surgical approach [1]. Currently, a large number of specialized centres around the world perform laparoscopy for urologic cancer [2, 3]. Nevertheless, local recurrence and port-site metastasis remain a concern [4].

Port-site metastases, though rare, have been extensively documented for other gynaecological and GI malignancies. When they occur, they often do so in the presence of advanced disease, but it is not uncommon for them to occur in isolation [5, 6]. Concern has been expressed that laparoscopic surgery might adversely affect the long-term outcomes by increasing the risk of port-site and peritoneal seeding.

The first known report of a port-site metastasis was by Dobronte and associates [7] in 1978. The authors reported implantation of malignant ovarian cystic adenoma in penetration sites of the pneumo-needle and trocar. Some specific procedures and tumors have been associated with a higher incidence of portsite metastasis or tumor seeding; however, the precise incidence of port-site metastasis and its aetiology and pathogenesis have not been well defined in urologic laparoscopy [8].

Port-site metastases is a multifactorial phenomenon with an as-yet undetermined incidence. Etiological factors include natural malignant disease behavior [9], host immune status [9], local wound factors [9], laparoscopy-related factors such as aerosolization of tumor cells (the use of gas, type of gas, insufflation and desufflation, and pneumoperitoneum) [9], and sufficient technical experience of the surgeons and operating team [9] (adequate laparoscopic equipment, skill, minimal handling of the tumor, surgical manipulation and wound contamination during instruments change, organ morcellation, and specimen removal) [9].

## 2. Materials and Methods

An electronic search of MEDLINE of the published literature up to 2010 was carried out using the combined MESH key words “port-site metastasis” and “Urology.”

 Duplicate references, as well as repeated references to the same data sets, were removed. The articles and case reports directly addressing port-site metastasis after laparoscopic surgery for urological malignancy were reviewed. Articles were selected and categorized by topic into incidence, aetiology, pathophysiology, and possible preventative measures.

## 3. Results

Table 1 showed the case reports found on MEDLINE search of the published literature up to 2010 recovered 51 for the MESH words “port-site metastasis” and “Urology.”

Etiological factor has been categorised in three main categories: tumour related, wound related, and surgical technique related. Surgical technique related factors have been categorised in two main categories: manipulation is the principal factor acting in tumour dissemination. Extraction of the surgical specimen is determined by the surgeon. The possible preventive measure has been categorised in two main categories: active measures and measures for reducing the risk of laparoscopic port-site metastasis in urological surgery.

## 4. Discussion

Laparoscopic surgery is rapidly gaining widespread acceptance among urologists, including extensive application in malignant conditions [9]. The incidence of tumour seeding in general laparoscopic surgery ranges from 0.8% to 21% (8, 9). However, most authors report an incidence of 0.5%, comparable to the rate for surgical wound metastases (0.8%–1.6%) in conventional open methods [9–11]. In recent years, several reports of port-site metastasis and tumor seeding have been published. Tsivian and Sidi [9] alone reported nine cases of port-site metastases after urologic laparoscopy, and Rassweiler and colleagues [10] published eight local recurrences observed in 1098 laparoscopic procedures for urologic malignancies. Recently, in an international survey of 19 urologic laparoscopic centres performing a total of 18,750 laparoscopic procedures for urologic malignancies, tumour seeding was reported in 13 cases (0.1%) [8].

Various theories tried to explain metastasis development at laparoscopic port site [12]. Factors can be divided into three categories: tumor related, wound related, and surgical technique related [4].

Tumor-related factors [8–10]: biological aggressiveness of the tumor, represented by grade and stage, could play a decisive role in possible tumor seeding determination, explaining why grade 2 and 3 transitional cell carcinomas represent the majority of port-site metastases in urological procedures [8–10].

Wound-related factors [11–17]: local and systemic immune response to the pneumoperitoneum has been suggested. Its physiopathological mechanism has yet to be completely defined. There is a tendency towards systemic preservation of the immune system and towards immune depression of the peritoneum during laparoscopic insufflation demonstrated by macrophage function alteration [11–17].

Surgical technique-related factors [9, 18–30]: manipulation is the principal factor acting in tumor dissemination. Extraction of the surgical specimen is determined by the surgeons [9, 18–30].

However, it is logical to assume that morcellation of the specimen increases tumor seeding [5, 6, 15]. The direct dissemination of tumor cells from contaminated material or from extraction with an unclosed bag is well documented [5, 6, 15]. The observance of a large number of tumor cells at excessively manipulated ports supports this hypothesis as well as observance of greater number of malignant cells at port sites used by the surgeon compared with those used by assistants [5, 6, 15].

The problem is influenced to some extent by surgeon and operating team experience [9, 31–37], and, therefore, it could be partially prevented [9, 31–37].

Port-site recurrence of tumour is a particular, and increasingly recognized [9, 31–37], drawback. certain measures have been suggested to prevent urologic port-site metastasis [9, 31–37], including (1) sufficient technical preparation, (2) avoidance of laparoscopic surgery if ascites ispresent [9, 31–37], (3) trocar fixation with avoidance of gas leakage along the trocar, (4) avoidance of tumor-boundary violation [9, 31–37], (5) cautious consideration of morcellation, (6) use of an impermeable bag if morcellation is done [9, 31–37], (7) use of a bag for intact specimen removal, (8) drainage placement if needed before abdominal deflation [9, 31–37], (9) povidone-iodine irrigation of the laparoscopic instruments, trocar, and port-site wounds [9, 31–37], and (10) suturing 10 mm trocar wounds [9, 31–37]. Povidone-iodine irrigation has been questioned, and peritoneal irritation secondary to this agent must not be underestimated [9, 31–37]. Regarding suggestion 10, Burns and coworkers [21] demonstrated on an animal model that portsite tumor implantation was significantly increased (*P* < 0.03) when only skin was closed compared with closure of all three layers [21]. The authors proved that closure technique may influence the rate of port-site tumor implantation [21]. For hand-assisted laparoscopic surgery, Chen and collaborators [38] recommend using a watertight bag model, not enlarging the surgical wound if there is resistance when extracting the surgical specimen and changing gloves before wound closure in order to avoid contamination with malignant cells [38]. Port-site metastasis in urological laparoscopic surgery is rare. Several factors have been associated with tumor seeding, but tumor grade and stage appear to have the greatest importance. Nevertheless, risk can be minimized by applying open surgery oncologic procedural norms [9, 18–30].

## 5. Conclusion

Port-site metastasis in urological laparoscopic surgery is rare. Multiple factors have been associated with tumour seeding, but tumour grade and stage appear to play a major role. Multiple methods have been described to reduce the risk of port-site metastasis. The incidence is comparable to the rate for surgical wound metastases.

## Figures and Tables

**Table 1:** The case reports found on MEDLINE.

Author	Procedure	Tumour type, stage, and grade	Number of cases
Stolla et al., 1994	Laparoscopic pelvic lymph node dissection	Bladder TCC pT3G2	1
Andersen et al.,1995	Transperitoneal laparoscopic bladder biopsy	Bladder TCC T1G2	1
Bangma et al., 1995	Laporoscopic pelvic lymph node dissection	PCa T3N1	1
Altieri et al., 1998	Laporoscopic pelvic lymph node dissection	Bladder TCC T3G2	1
Ahmed et al., 1998	Laparoscopic nephrectomy	Kidney TCC T3G3-G4	1
Otani et al., 1999	Laparoscopic nephrectomy	Incidental finding of TCC, G3 within tuberculous atrophic kidney	1
Fentie et al., 2000	Laparoscopic nephrectomy	RCC T3N0G4	1
Landman and Clayman, 2001	Laparoscopic nephrectomy	RCC T1N0G2	1
Castilho et al., 2001	Laparoscopic nephrectomy	RCC T1N0G2	1
Wang et al., 2002	Laparoscopic cystectomy	Incidental finding of SCC in ovarian dermoid cyst	1
Chen et al., 2003	Laparoscopic nephrectomy (hand assisted)	RCC T2N0M0	1
Rassweiler et al., 2003	Laparoscopic adrenalectomy	Small-cell lung carcinoma adrenal metastasis	1
	Laparoscopic retroperitoneal lymph node dissection	NA	1
Saraiva P et al., 2003	Laparoscopic adrenalectomy	Metastatic melanoma of adrenal gland. Grade unavailable	1
Matsui et al., 2004	Laparoscopic retroperitoneal nephroureterectomy	SCC pT3N0M0	1
Iwamura et al., 2004	Laparoscopic retroperitoneal nephrectomy	RCC T1bN0M0	1
Micali et al., 2004	Laparoscopic Adrenalectomy	Lung metastases pT4/G3 (3); Adrenocortical Ca-grade and stage NA (1)	4
	Laporoscopic pelvic lymph node dissection	Squamous penile Ca	1
	Laparoscopic retroperitoneal lymph node dissection	Nonseminomatous Germ Cell Tumor	1
	Laparoscopic simple nephrectomy	Incidental TCC in each instance—pT1/G2; pT1/G3; pT2/G3; NA	4
	Laparoscopic nephroureterectomy	pT3/G3	3
Naderi et al., 2004	Laporoscopic nephroureterectomy	Kidney TCC cT1N0M0	1
Chueh et al., 2004	Laporoscopic bilateral nephroureterectomy	Grade 2 renal TCC with pelvic muscular invasion and bladder metastasis	1
Porpiglea et al., 2004	Laparoscopic adrenalectomy	Adrenal metastasis from nonsmall cell lung carcinoma	1
El-Tabey and Shoma, 2005	Laparoscopic cystectomy (robot-assisted)	Bladder TCC T3bN0M0G3	1
Kobori et al., 2005	Laparoscopic nephrectomy	Papillary adenocarcinoma of pelvis. Stage and grade unavailable	1
Dhobada et al., 2006	Laparoscopic nephrectomy	RCC T2N0M0G3	1
Manabe et al., 2007	Laparoscopic nephroureterectomy	Upper tract transitional cell carcinoma without distant metastases	1
Muntener et al., 2007	Laparoscopic radical nephroureterectomy	Upper tract TCC. Stage T1, high grade	1
Castillo and Vitagliano 2008	Laparoscopic partial nephrectomy	RCC T1N0M0G3	1
	Laparoscopic retroperitoneal lymph node dissection	Mixed germ cell tumor T3N0M0	1
Cresswell et al., 2008	Laporoscopic retroperitoneal lymph node dissection	Stage 1 nonseminomatous germ cell tumour. Grade NA	1
Segawa et al., 2008	Laparoscopic nephroureterectomy and cystectomy	Invasive bladder cancer with bone metastasis. Grade NA	1
Spermon and Witjes 2008	Laparoscopic retroperitoneal lymph node dissection	Stage IIb non seminomatous germ cell tumour (Histology-yolk sac and teratoma elements)	1
Greco et al., 2009	Laparoscopic partial nephrectomy	Renal clear cell papillary carcinoma pT1a, high grade	1
Yasuda et al., 2009	laparoscopic nephroureterectomy	Upper urinary tract carcinoma. T2N0M0 Grade 2 > 3	1
Huang et al., 2010	Laparoscopic radical cystectomy and pelvic lymph node dissection	NA	1

Pca = prostate cancer, RCC = Renal cell carcinoma, SCC cell carcinoma, NA = not available.
